# Neonicotinoid detection in wild turkeys (*Meleagris gallopavo silvestris*) in Ontario, Canada

**DOI:** 10.1007/s11356-018-2093-0

**Published:** 2018-04-27

**Authors:** Amanda M. MacDonald, Claire M. Jardine, Philippe J. Thomas, Nicole M. Nemeth

**Affiliations:** 1 Department of Pathobiology, Ontario Veterinary College, University of Guelph, Guelph, ON NIG 2W1 Canada; 2 Canadian Wildlife Health Cooperative, Ontario Veterinary College, University of Guelph, Guelph, ON NIG 2W1 Canada; 3 Environment and Climate Change Canada, Science and Technology Branch, National Wildlife Research Center, Ottawa, ON K1A 0H3 Canada; 4 Present Address: Southeastern Cooperative Wildlife Disease Study, University of Georgia, Athens, GA 30602 USA

**Keywords:** Bioaccumulation, Birds, Insecticides, Neonicotinoids, Non-target species, Pesticides, Treated seeds, Wild Turkey

## Abstract

The use of neonicotinoid insecticides in agriculture is now recognized for the health risks it poses to non-target wildlife, with associated honey bee mortality especially concerning. Research directed toward the presence and effects of these pesticides on terrestrial vertebrates that consume neonicotinoid-coated seeds, such as wild turkeys (*Meleagris gallopavo silvestris*), is lacking. This study used liquid chromatography attached to a tandem mass spectrometer to assess the liver from 40 wild turkeys for neonicotinoid and other pesticide residues and compared detected levels of these contaminants across the southern Ontario, Canada. Nine (22.5%) wild turkeys had detectible levels of neonicotinoid residues—clothianidin in eight, and thiamethoxam in three. Two (5.0%) of these turkeys had detectable levels of both clothianidin and thiamethoxam. Fuberidazole was detected in two (5.0%) wild turkeys. The highest level of thiamethoxam detected was 0.16 ppm, while clothianidin was detected at 0.12 ppm, and fuberidazole at 0.0094 ppm. Knowledge of exposure in free-ranging wildlife is critical for better understanding the effects of neonicotinoids on wildlife health; thus, these data help establish baseline data for southern Ontario wild turkeys and provide context for reference values in future analyses.

## Introduction

Neonicotinoid insecticides (NNIs) have become the most widely used insecticides in the world (Schaafsma et al. 2015). Commonly used in agriculture, they are applied as various formulations including as foliage sprays, seed coating, and soil treatments. Of NNIs used globally, including those used on many large-acreage crops in southern Ontario (e.g., corn, soy, grains, dry beans, and canola), 60% are utilized as seed coatings (Jeschke et al. 2011; OMNRF 2017). NNIs are systemic insecticides, taken up by the plant following application and distributed systemically through plant tissues as it grows. They act by affecting the central nervous system of insects, causing over-excitation of nerve synapses, followed by paralysis and eventually death (Fishel 2013). Recently, these insecticides have been recognized for the risks they pose to non-target wildlife, including as a potential factor driving colony collapse disorder in honey bees (Farooqui 2013). However, little attention has been paid to higher trophic biota, including terrestrial vertebrates.

Wild turkeys (*Meleagris gallopavo*), and other avian species such as gray partridges (*Perdix perdix*) and pigeons (*Columba palumbus*, *C. livia*, *and C. oenas*), readily ingest treated corn or soya seeds (Mineau and Palmer 2013; Millot et al. 2017); in fact, depending on food availability, these resources comprise a significant portion of the wild turkey’s diet (OMNRF 2007). These seeds can contain some of the highest concentrations of NNIs (Gibbons et al. 2015), making them of particular concern because of their availability to birds and the potential for repeat or ongoing exposure. A single corn kernel is typically treated with approximately 1 mg of active ingredient (Rexrode et al. 2003) and consumption of just one imidacloprid-treated corn seed, or a few clothianidin- or thiamethoxam-treated seeds, could be lethally toxic to a bird the size of a blue jay (Mineau and Palmer 2013). The persistence of NNIs in the environment as well as their potential ill-effects on non-target species remains unclear. Recently, there has been a great deal of public and political controversy and media coverage regarding the use and associated risks of NNIs to honey bee health and mortality. There has also been growing concern among natural resource managers, conservationists, and hunters about whether NNI use may be linked to poor reproductive output of wild turkeys and potential bioaccumulation of NNIs in wild turkey meat intended for human consumption.

The present study was conducted to address knowledge gaps and the concerns described above. Samples originated from healthy-appearing, hunter-harvested wild turkeys from across southern Ontario. The objectives were to (1) assess wild turkey liver tissues for NNIs and other potential environmental contaminants and (2) compare levels of detected contaminants across the geographic area of the study site.

## Materials and methods

**Fig. 1:**
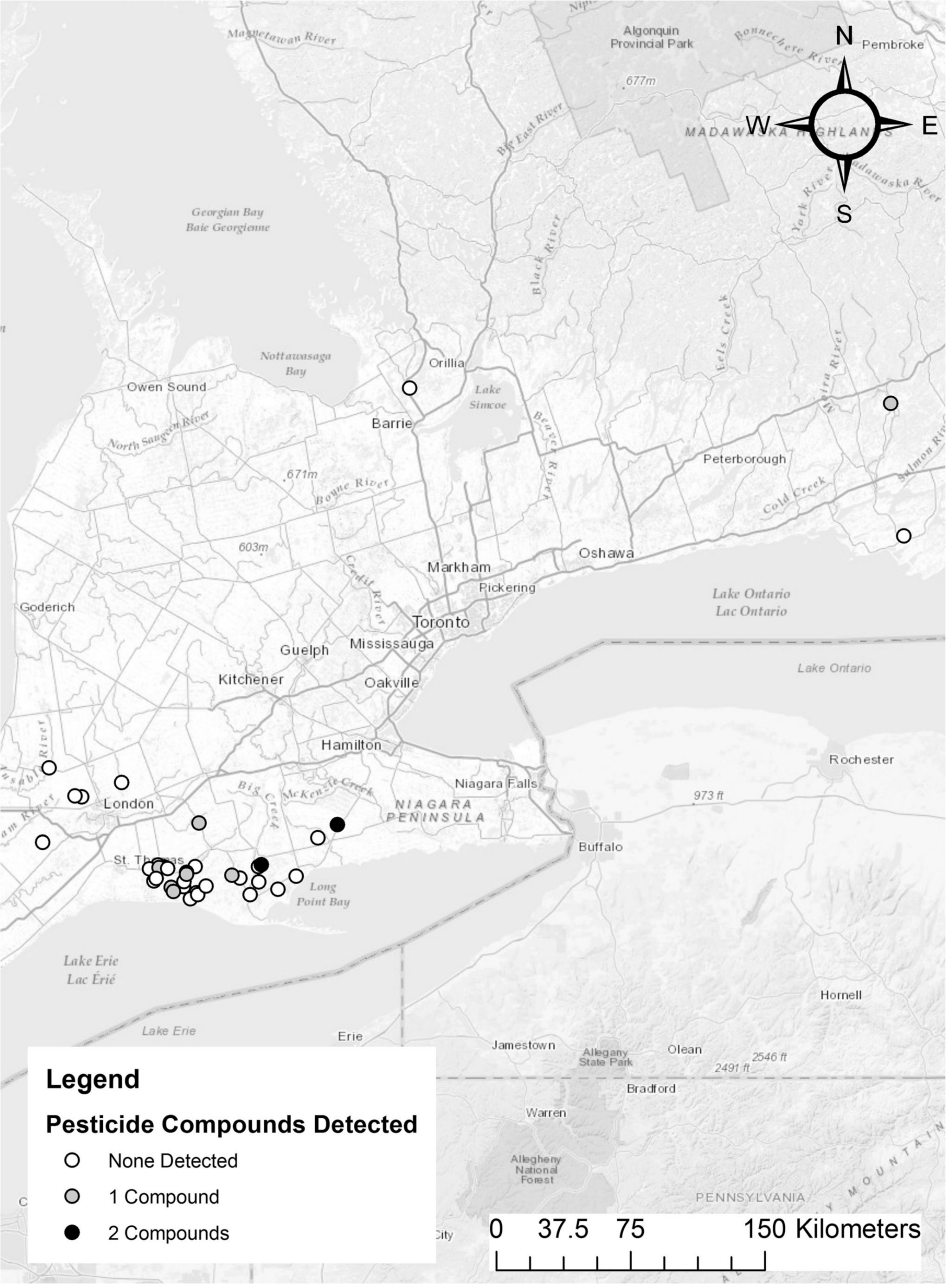
Map depicting the locations and pesticide compounds detected among 40 hunter-harvested wild turkeys collected during the 2015 spring hunt (April–May) in Ontario, Canada

During the 2015 Ontario spring hunting season (April–May), 147 hunter-harvested wild turkey carcasses were collected (Wildlife Scientific Collector’s Authorization from the Ontario Ministry of Natural Resources and Forestry, #1079555) from across southern Ontario. Carcasses were stored at − 20 °C and thawed at 4 °C prior to sample collection. Liver samples from each turkey were collected and stored at − 80 °C until testing. Forty samples were selected to represent regions across the collection range (Fig. 1) and were sent for pesticide residue analysis at Laboratory Services, Agriculture and Food Laboratory, University of Guelph*,* Guelph, Ontario. The testing proceeded using liquid chromatography attached to a tandem mass spectrometer (LC-MS/MS). The TOPS-142 screening procedure included the analysis and quantification of 142 different pesticide compounds, including seven neonicotinoids (Wang and Leung 2009).

Extraction of pesticides from wet turkey livers was performed using the QuEChERS method (Anastassiades et al. 2003). Briefly, each sample was extracted using 1% acetic acid in acetonitrile and liquid phases were partitioned using sodium acetate and anhydrous magnesium sulfate. The supernatant was cleaned with methanol and 0.1 M ammonium acetate. Sample extracts were analyzed using LC-MS/MS operated in electrospray ionization mode.

## Results

Of 40 wild turkey livers tested, all were male (due to hunter-harvest regulations), 3 were juveniles, and 37 were adults. NNI compounds were detected by LC-MS/MS in liver samples from 17 wild turkeys (i.e., 43% of samples analyzed) at levels approaching the lower detection limit. In 6 of these samples, levels were higher than the lower detection limit but still below quantification limits (“Appendix” Table 2). Nine of 40 (22.5%) adult wild turkeys had detectible levels of NNI residues, clothianidin in eight, and thiamethoxam in three. Two (5.0%) of these turkeys had detectable levels of both clothianidin and thiamethoxam. Fuberidazole (a fungicide used as a seed treatment for cereals) was detected in two (5.0%) wild turkeys. The highest levels detected for each compound were 0.16 ppm of thiamethoxam, 0.12 ppm of clothianidin, and fuberidazole at 0.0094 ppm (Table 1).

**Table 1:** Summary of detectible levels of pesticide residues in livers of hunter-harvested wild turkeys (*n* = 40) in April–May 2015, from Ontario, Canada

Pesticide	Main use	No. of turkeys (%)	MDL (ppm)	Range (ppm)
Clothianidin	Insecticide	8 (20.0)	0.001	0.0086–0.1200
Thiamethoxam	Insecticide	3 (7.5)	0.001	0.0110–0.1600
Fuberidazol	Fungicide	2 (5.0)	0.0005	0.0077–0.0094

*MDL* minimum detection limit

## Discussion

Neonicotinoids are insecticides used worldwide in agriculture as seed treatments on crops such as corn and soya (Garthwaite et al. 2003; Jeschke et al. 2011). This class of pesticide includes acetamiprid, clothianidin, dinotefuran, imidacloprid, nitenpyram, nithiazine, thiacloprid, and thiamethoxam. Imidacloprid, clothianidin, and thiamethoxam are the most common NNIs applied for agricultural use in Ontario (Somers and Chung 2014). Wild turkeys in Ontario and other regions live within concentrated areas used for agriculture and often rely on croplands for supplemental feeding to survive, particularly in winter months (Porter et al. 1980; Vander Haegen et al. 1989). Thus far, NNIs have been best known for the adverse effects they cause in honey bees. Mass die-offs, and subsequent declines in bee population numbers, have garnered widespread attention and concern in recent years because of the importance of bees in providing a vital ecosystem service, namely, pollination (Whitehorn et al. 2012; Godfray et al. 2015; Rundlöf et al. 2015).

Pesticides, including the NNIs clothianidin and thiamethoxam, were detected in the liver of nearly half of wild turkeys tested. These turkeys were likely exposed to NNIs by consuming pesticide-coated seeds during crop sowing that spring, as treated seeds were observed in the crops of several birds at the time of necropsy. Although accumulated residues were low, evidence is mounting that non-target avian species, such as partridges, pigeons, and quail (*Colinus virginianus*, *Coturnix japonica*), are exposed through the consumption of these coated seeds (Mineau and Palmer 2013; Millot et al. 2017). Recently, concentrations up to 0.067 mg/kg of thiamethoxam/clothianidin were reported in failed eggs of gray partridge known to frequent pesticide-treated cereal fields in north-central France (Bro et al. 2016). Very little published information is available on fuberidazole in non-target species; however, it was suspected to have played a role in the morbidity observed in pheasants feeding on treated wheat within a game farm in the UK (Laing 2001).

Most experimental pesticide toxicity studies are limited to observations of acute toxicity in laboratory rats, even though birds are often more susceptible than rats to pesticide toxicity. These laboratory assessments often target single compounds, when in reality, non-target wildlife species are exposed to complex mixtures of pesticides/contaminants with synergistic and/or inhibitory effects. Such laboratory studies also neglect to consider species-specific sensitivities to single compounds, or complex mixtures that could ultimately impair whole populations. For example, clothianidin, which was detected in 20% of wild turkeys in the present study, is considered far less toxic to rats (LD_50_ > 5000 mg/kg) compared to Japanese quail (423 mg/kg) and northern bobwhite quail (> 2000 mg/kg). The LD_50_ of imidacloprid in rats ranges from 379 to 648 mg/kg (or ppm), but this dose is much lower for birds: 14 mg/kg for gray partridge, 31 mg/kg for Japanese quail, and 152 mg/kg for northern bobwhite quail (SERA 2005; Anon 2012; Rose 2012; Mineau and Palmer 2013). Currently available toxicity data often disregard the chronic effects of exposure, which may occur at lower concentrations and over longer periods of time in free-ranging wildlife or other animals. For example, a dose equivalent to 0.10% of a neonicotinoid-coated corn seed ingested daily during the egg-laying season can adversely affect reproduction in birds (Mineau and Palmer 2013).

Although not detected in the present study, imidacloprid is considered the most toxic NNI in birds (EPA 2016), although toxicity varies across species. For example, a study involving pigeons and partridges found dead in a barn following exposure to coated seeds showed hepatic toxicity levels of imidacloprid ranging from 1.0–1.6 μg/g (ppm) in partridges and up to 3.1 μg/g in pigeons (Berney et al. 1999). Recent experimental research on the migratory white-crowned sparrow (*Zonotrichia leucophrys*) in Saskatchewan, Canada, showed that delays in and impaired orientation during migration, loss of body mass, decreased reproduction efforts, and potentially increased mortality are possible outcomes after consuming approximately 4 imidacloprid-treated canola seeds, or just 0.2 treated corn seeds (i.e., dosage of 4.1 μg imidacloprid/g bw/day, equivalent to 10% of the LD_50_ of house sparrows; Eng et al. 2017).

Additional research is required to determine the chronic health and reproductive effects on wild turkeys and other wildlife that may occur with repeated exposure and ingestion of NNI-coated seeds. For example, knowledge gaps exist on the timing and duration of exposure, the rate at which these chemicals are metabolized in birds, and what proportion of the ingested material reaches the liver. Such information will require both field studies to understand wildlife feeding habits and behavior and experimental studies to delineate toxic levels and associated clinical effects. It should be noted that studies such as ours also carry inherent sampling biases. First, we relied on sampling of hunter-harvested wild turkeys, which tend to be skewed toward larger, healthy-appearing male birds. Also, wild turkeys that may be suffering from morbidity or mortality associated with NNI ingestion are less likely to be recovered and tested, as they may hide in vegetation prior to death or be killed and consumed by predators.

Little is known about NNI persistence and impacts on non-target avian species in agricultural landscapes. As of July 1, 2015, new regulatory requirements came into effect for the sale and use of NNI-treated seeds in Ontario. These requirements support reducing the use of imidacloprid, thiamethoxam, and clothianidin on specific crops (corn and soybean) planted with NNI-treated seeds by 80% by 2017; however, only an estimated 25% reduction has been reported based on 2014 baseline data (MOECC 2015). Knowledge of chronic and acute bird exposure, particularly in farmland birds, should be a first step in understanding the effects of NNIs on the health of birds and other wildlife. These data are important to serve as baseline data for southern Ontario wild turkeys and to provide context for reference values in future analyses.

## Appendix

**Table 2:** Pesticide residue testing results from livers of 40 hunter-harvested wild turkeys in April–May 2015, from Ontario, Canada

	Neonicotinoid (ppm)								Other (ppm)	
Sample	Acetamiprid	Clothianidin	Dinotefuran	Imidacloprid	Nitenpyram	Thiabendazole	Thiacloprid	Thiamethoxam	Fuberidazol	Mandipropamid
MDL (ppm)	0.001	0.001	0.002	0.001	0.001	0.0005	0.001	0.001	0.0005	0.0005
1	N.D.	N.D.	N.D.	N.D.	N.D.	< MDL	N.D.	N.D.	N.D.	N.D.
2	N.D.	N.D.	N.D.	N.D.	N.D.	N.D.	N.D.	N.D.	N.D.	N.D.
3	N.D.	N.D.	N.D.	N.D.	N.D.	< MDL	N.D.	N.D.	N.D.	N.D.
4	N.D.	N.D.	N.D.	N.D.	N.D.	N.D.	N.D.	N.D.	N.D.	N.D.
5	N.D.	0.11	N.D.	N.D.	N.D.	N.D.	N.D.	N.D.	N.D.	N.D.
6	N.D.	N.D.	N.D.	N.D.	N.D.	N.D.	N.D.	N.D.	< MDL	N.D.
7	N.D.	N.D.	N.D.	N.D.	N.D.	N.D.	N.D.	N.D.	N.D.	N.D.
8	N.D.	N.D.	N.D.	N.D.	N.D.	N.D.	N.D.	N.D.	N.D.	N.D.
9	N.D.	N.D.	N.D.	N.D.	N.D.	N.D.	N.D.	N.D.	N.D.	N.D.
10	N.D.	0.0091	N.D.	N.D.	N.D.	N.D.	N.D.	< MDL	N.D.	N.D.
11	N.D.	< MDL	N.D.	N.D.	N.D.	N.D.	N.D.	N.D.	N.D.	N.D.
12	N.D.	< MDL	N.D.	N.D.	N.D.	N.D.	N.D.	0.011	< MQL	N.D.
13	N.D.	N.D.	N.D.	N.D.	N.D.	N.D.	N.D.	N.D.	N.D.	N.D.
14	N.D.	< MDL	N.D.	N.D.	N.D.	N.D.	N.D.	N.D.	N.D.	N.D.
15	N.D.	0.069	N.D.	N.D.	N.D.	N.D.	N.D.	N.D.	N.D.	N.D.
16	N.D.	< MDL	N.D.	N.D.	N.D.	< MDL	N.D.	N.D.	N.D.	N.D.
17	N.D.	< MQL	N.D.	N.D.	N.D.	N.D.	N.D.	< MQL	N.D.	N.D.
18	N.D.	N.D.	N.D.	N.D.	N.D.	N.D.	N.D.	N.D.	N.D.	< MDL
19	N.D.	< MDL	N.D.	N.D.	N.D.	N.D.	N.D.	N.D.	N.D.	N.D.
20	N.D.	< MQL	N.D.	N.D.	N.D.	N.D.	N.D.	N.D.	N.D.	N.D.
21	N.D.	N.D.	N.D.	N.D.	N.D.	N.D.	N.D.	N.D.	N.D.	N.D.
22	N.D.	< MDL	N.D.	N.D.	N.D.	N.D.	N.D.	N.D.	< MDL	N.D.
23	N.D.	N.D.	N.D.	N.D.	N.D.	N.D.	N.D.	N.D.	N.D.	N.D.
24	N.D.	0.0089	N.D.	N.D.	N.D.	< MDL	N.D.	< MQL	N.D.	N.D.
25	N.D.	N.D.	N.D.	N.D.	N.D.	N.D.	N.D.	N.D.	N.D.	N.D.
26	N.D.	N.D.	N.D.	N.D.	N.D.	N.D.	N.D.	N.D.	N.D.	N.D.
27	N.D.	0.026	N.D.	N.D.	N.D.	N.D.	N.D.	N.D.	N.D.	N.D.
28	N.D.	N.D.	N.D.	N.D.	N.D.	N.D.	N.D.	N.D.	N.D.	N.D.
29	N.D.	N.D.	N.D.	N.D.	N.D.	N.D.	N.D.	N.D.	N.D.	N.D.
30	N.D.	0.023	N.D.	N.D.	N.D.	N.D.	N.D.	0.16	N.D.	< MDL
31	N.D.	N.D.	N.D.	N.D.	N.D.	N.D.	N.D.	N.D.	N.D.	N.D.
32	N.D.	N.D.	N.D.	N.D.	N.D.	N.D.	N.D.	N.D.	N.D.	N.D.
33	N.D.	0.0086	N.D.	N.D.	N.D.	N.D.	N.D.	0.016	N.D.	N.D.
34	N.D.	N.D.	N.D.	N.D.	N.D.	N.D.	N.D.	N.D.	N.D.	N.D.
35	N.D.	< MDL	N.D.	N.D.	N.D.	N.D.	N.D.	N.D.	N.D.	N.D.
36	N.D.	0.12	N.D.	N.D.	N.D.	< MDL	N.D.	N.D.	N.D.	N.D.
37	N.D.	N.D.	N.D.	N.D.	N.D.	N.D.	N.D.	N.D.	0.0094	N.D.
38	N.D.	N.D.	N.D.	N.D.	N.D.	< MQL	N.D.	N.D.	0.0077	N.D.
39	N.D.	N.D.	N.D.	N.D.	N.D.	N.D.	N.D.	N.D.	N.D.	N.D.
40	N.D.	N.D.	N.D.	N.D.	N.D.	N.D.	N.D.	N.D.	N.D.	N.D.

*N.D.* not detected, *< MDL* less than minimum detection limits, *< MQL* less than minimum quantification limits
